# A Bifactor Measure of Societal Stigma Toward Eating Disorders and Obesity: Scale Development and Validation

**DOI:** 10.3390/ijerph23030399

**Published:** 2026-03-20

**Authors:** Carlos Suso-Ribera, Laura Díaz-Sanahuja, Macarena Paredes-Mealla, Sara Marsal, Miriam Almirall

**Affiliations:** 1 Rheumatology Research Group, Vall d’Hebron Research Institute, 08035 Barcelona, Spain; sara.marsal@vhir.org (S.M.); miriam.almirall@vallhebron.cat (M.A.); 2 Faculty of Health Sciences, Universidad Europea de Canarias, 38300 La Orotava, Spain; 3 Faculty of Psychology and Speech Therapy, Universidad de Valencia, 46010 Valencia, Spain; laura.diaz-sanahuja@uv.es; 4 Hospital Provincial Foundation, 12006 Castellon, Spain; dparedes@uji.es; 5 Department of Rheumatology, Hospital Universitari Vall d’Hebron, 08035 Barcelona, Spain

**Keywords:** eating disorders, obesity, stigma, scale development, psychometric validation

## Abstract

**Highlights:**

**Public health relevance—How does this work relate to a public health issue?**

- Societal stigma toward eating disorders and obesity is a modifiable public health risk factor linked to psychological distress, reduced help-seeking, and social and healthcare inequalities.
- By documenting the structure and distribution of stigma in a Spanish-speaking population, this study situates stigma as a population-level issue relevant to prevention, early detection, and health equity.

**Public health significance—Why is this work of significance to public health?**

- The study advances public health measurement by integrating contemporary stigma frameworks into a psychometrically robust instrument suitable for large-scale population research.
- Evidence on how stigma varies by age, gender, and experiential factors strengthens the scientific basis for understanding stigma as a social determinant of health.

**Public health implications—What are the key implications or messages for practitioners, policy makers and/or researchers in public health?**

- Findings support the inclusion of stigma assessments in public health surveillance and evaluation of prevention and health promotion strategies related to eating disorders and obesity.
- Public health actions should move beyond individual responsibility narratives and address stigma through structural, educational, and media-level interventions that promote compassionate and equitable care.

**Abstract:**

Background: Societal stigma toward eating disorders and obesity remains pervasive and is associated with psychological distress, maladaptive eating behaviors, reduced help-seeking, and barriers to care. Despite its documented impact, comprehensive and psychometrically robust instruments to assess stigma—particularly in Spanish-speaking populations—are scarce. This study aimed to develop and validate a multidimensional measure of societal stigma toward eating disorders and obesity in Spain, grounded in contemporary stigma frameworks. Methods: A cross-sectional observational study was conducted in a large community sample recruited online (*N* = 2121). An initial pool of stigma-related items was developed based on theoretical and empirical literature and refined through expert content validation. Psychometric evaluation included item screening, exploratory factor analysis (EFA), confirmatory factor analysis (CFA), bifactor modeling, and reliability assessment. The sample was randomly split for EFA (*n* = 988) and CFA (*n* = 658). Associations between stigma scores and sociodemographic and experiential variables were examined. Results: The final 36-item instrument demonstrated excellent psychometric properties. Bifactor analyses supported an essentially unidimensional structure dominated by a strong general stigma factor, with secondary content-specific dimensions (e.g., legitimacy, personal responsibility, visibility, and treatment beliefs). The theory-driven bifactor model showed excellent fit (CFI = 0.991; TLI = 0.990; RMSEA = 0.024). The general factor exhibited high reliability (ω_h_ = 0.87). Higher stigma was observed among men, older participants, and individuals without personal or familial experience of eating disorders or obesity. Conclusions: This study provides a reliable and theoretically grounded instrument for assessing societal stigma toward eating disorders and obesity in Spain. The scale enables systematic research on stigma and offers a valuable tool for public health surveillance, intervention development, and evaluation of anti-stigma initiatives aimed at promoting compassionate and equitable care.

## 1. Introduction

Food- and body-related conditions are highly visible targets of societal scrutiny and moral judgment, a phenomenon that has intensified in recent decades alongside the rise of social media, pervasive diet culture, and culturally mediated beauty ideals [1,2,3,4]. Digital platforms such as Instagram, Facebook, and TikTok expose users—particularly adolescents and young adults—to idealized body images and prescriptive “healthy lifestyle” narratives that frequently moralize food choices, body weight, and self-control [3,4]. This environment promotes social comparison, body dissatisfaction, and psychological distress, while reinforcing a moral hierarchy of bodies in which thinness, discipline, and normative eating practices are valorized and deviation is stigmatized [1,2]. These moralized social norms contribute to pervasive stigma toward individuals with eating disorders and obesity, shaping public attitudes, social inclusion, and engagement with healthcare systems [3,4]. Individuals with eating disorders are often perceived as attention-seeking, manipulative, or “not ill enough,” particularly in the case of binge-eating presentations [5], whereas people with obesity are frequently framed as lazy, undisciplined, or personally responsible for their condition [6]. Such narratives obscure the complex biological, psychological, and socio-environmental determinants of both conditions, intensify psychological distress, and reduce engagement with evidence-based care [7,8].

Empirical evidence consistently demonstrates that weight stigma—whether experienced, anticipated, or internalized—is associated with maladaptive eating cognitions and behaviors, including binge eating, dietary restraint, unhealthy weight control practices, drive for thinness, and body dissatisfaction [9]. These associations have been observed across demographic groups and clinical presentations, underscoring stigma as a transdiagnostic risk factor for disordered eating and impaired well-being. Importantly, stigma is not only a consequence of misinformation but is closely linked to gaps in eating disorder mental health literacy, including persistent myths regarding causes, severity, and recovery. For example, adolescents with lower knowledge about eating disorders endorse higher stigma and more stereotypical beliefs, particularly among males [10], highlighting the role of knowledge deficits in sustaining stigmatizing attitudes. These findings underscore the profound impact of stigma on disordered eating and highlight the urgent need to address stigma at structural, interpersonal, and individual levels.

Conceptual frameworks are essential for clarifying and measuring stigma systematically. The Mental Illness Stigma Framework (MISF) emphasizes the importance of precise definitions and consistent terminology to capture stigma mechanisms such as stereotypes, prejudice, discrimination, and the perspectives of stigmatized individuals (experienced, anticipated, and internalized stigma) [11]. Similarly, the Health Stigma and Discrimination Framework conceptualizes stigma as a multilevel phenomenon operating across structural, interpersonal, and individual domains, incorporating beliefs about legitimacy, personal responsibility, visibility, chronicity, and recovery potential [12]. These frameworks highlight how societal norms, power relations, and intersecting social identities—such as gender, socioeconomic status, and body size—shape stigma and its consequences for help-seeking, healthcare access, and social participation. In this context, eating disorders and obesity are increasingly understood as complex conditions influenced by biological, psychological, and socio-environmental factors, as well as by broader systems of inequality and moral regulation of bodies [13].

In Spain, eating disorders predominantly affect adolescents and young adults, with prevalence estimates ranging from 3% to 6% in community samples and consistently higher rates among females than males [14,15,16]. Although onset typically occurs in adolescence, these disorders often persist into adulthood, with evidence of ongoing incidence and chronicity [14,15,17]. Overweight and obesity also affect a substantial proportion of the Spanish population, with excess weight present in 63.7% of men and 48.4% of women, and obesity affecting approximately one in five adults [18]. These conditions carry significant long-term health risks [4], are not mutually exclusive, and often co-occur—particularly in the context of binge-eating disorder and bulimia nervosa—reflecting shared neurobiological vulnerabilities, difficulties in emotion regulation, and socio-environmental risk factors [19,20]. Shared mechanisms—including dysregulated appetite signaling, compulsive eating patterns, altered reward processing, and body image dissatisfaction—interact with social influences such as weight-based teasing and stigmatizing media representations, amplifying both clinical severity and exposure to stigma [19,21,22,23]. Historically, the separation of eating disorders as psychiatric conditions and obesity as a lifestyle issue has further reinforced moralized distinctions that perpetuate stigma [19,21].

Despite robust evidence of the harmful effects of stigma, systematic tools to assess societal stigma toward eating disorders and obesity—particularly in Spanish-speaking populations—remain scarce [5]. Existing instruments are often unidimensional, narrowly focused, or insufficiently validated [5,11,24]. Guided by contemporary stigma frameworks and emerging evidence highlighting the role of misinformation, moralization, and structural inequities, the present study aimed to develop and validate a psychometrically robust instrument to assess societal stigma toward eating disorders and obesity in Spain. By capturing stigma comprehensively at the societal level, this tool seeks to inform research, public health strategies, and interventions designed to reduce blame, challenge myths, and promote more compassionate and equitable responses to eating- and weight-related conditions. Unlike existing instruments, the present scale integrates multiple stigma domains within a unified bifactor framework and is specifically developed for Spanish-speaking populations, enabling comprehensive and population-level assessment of societal stigma toward eating disorders and obesity.

## 2. Material and Methods

### 2.1. Study Design

This study employed a cross-sectional observational design to develop and validate a multidimensional framework for stigma related to eating disorders. The methodology closely followed a prior stigma scale developed by the authors in schizophrenia research, ensuring conceptual and analytical consistency while adapting items to the phenomenology and social representations specific to eating disorders.

The primary aim was to examine content validity and internal structure of a set of stigma-related statements organized into theoretically grounded domains. The study was designed to support psychometric analyses (e.g., factor analyses) and explore associations between stigma dimensions and relevant sociodemographic and experiential variables.

### 2.2. Participants and Sampling

Participants were recruited from the general population using online convenience sampling. A virtual snowball strategy was implemented, combining dissemination via social media and paid online advertisements. Participation was voluntary, with no financial or material compensation offered. The target sample size was guided by established recommendations for scale development and factor analysis, which suggest a minimum of 5–10 participants per item and favor large samples (e.g., *N* > 1000) to ensure stable parameter estimation and robust factor solutions [25].

Inclusion criteria required participants to be 18 years or older, consistent with the legal age of majority in Spain, reside in the country of survey administration, have sufficient Spanish proficiency to complete the questionnaire, and signing an informed consent form. Unlike studies focusing exclusively on public stigma, individuals with lived experience of eating disorders or weight-related conditions were included, enabling examination of stigma across different levels of personal experience.

All responses were collected anonymously. The study protocol adhered to ethical standards for human research, and electronic informed consent was obtained prior to survey completion.

### 2.3. Measures

#### 2.3.1. Eating Disorders Stigma Item Set

The eating disorders stigma item set was developed through a multi-stage process grounded in theoretical and empirical literature on stigma, eating disorders, obesity, and weight-related bias. Item development mirrored the authors’ prior schizophrenia stigma research to ensure methodological continuity.

##### Conceptual Framework and Category Definition

Based on an integrative literature review, items were created to tap into seven conceptually distinct yet related domains of societal stigma toward eating disorders and obesity present in the literature:

- Sociodemographic Stereotypes: Beliefs related to age, gender, socioeconomic status, seasonality, professions, or perceived rarity of eating disorders. For example, eating disorders are often perceived as primarily affecting young, female, and middle- to upper-class individuals, which can lead to under-recognition and delayed treatment in males, older adults, or individuals from other socioeconomic backgrounds [26].
- Severity and Legitimacy: Stigma can involve trivialization, normalization, minimization, or questioning of the seriousness, authenticity, or medical legitimacy of eating disorders and obesity. For example, individuals with BED may be perceived as “not really sick” or as exaggerating their struggles, while obesity is often dismissed as a lifestyle choice rather than a complex health condition, which can reduce empathy and delay engagement with care [5,6].
- Visibility and Detectability: Beliefs that eating disorders or obesity are easily observable or visually obvious can contribute to underdiagnosis and delayed help-seeking. For instance, anorexia nervosa is often stereotyped as visibly thin, which can lead to misrecognition of EDs in individuals with less typical presentations or higher body weight [27].
- Chronicity and Recovery: Stigmatizing narratives frequently polarize recovery expectations, portraying eating disorders as either inevitably chronic and untreatable or unrealistically easy to overcome. For example, bulimia nervosa may be perceived as a self-inflicted habit that can be controlled with willpower, whereas anorexia nervosa is sometimes seen as a lifelong, unchangeable condition, fostering hopelessness among patients and caregivers [5].
- Personal Responsibility and Moral Judgment: Stigma often manifests as blame or moral judgment, framing individuals as weak, lacking willpower, or morally deficient. This overlaps with weight stigma, where people with obesity are frequently characterized as lazy or undisciplined, and those with eating disorders are seen as manipulative or seeking attention [28,29].
- Treatment, Resource Allocation, and Social Response: Stigma operates at structural, interpersonal, and institutional levels, affecting secrecy, attitudes toward treatment, public spending, empathy, family burden, and societal management of eating- and weight-related conditions. For example, individuals with obesity or binge eating disorder may face reduced access to specialized care, while family members may experience judgment or social blame for their relative’s condition [30].
- Etiological Beliefs (Biological, Psychological, Social): Beliefs about the causes of eating disorders and obesity shape stigmatizing attitudes. For instance, perceiving eating disorders as solely a psychological weakness or obesity as the result of poor lifestyle choices can reinforce blame, whereas awareness of biological or socio-environmental contributors may reduce stigma and promote empathy [31].

#### 2.3.2. Item Development and Content Validation

An initial pool of statements was generated for each category as declarative items reflecting societal beliefs, myths, and assumptions about eating disorders and obesity. Vignette-based approaches were avoided to capture generalized beliefs rather than responses constrained to hypothetical cases.

Content validity was assessed by two external expert judges in clinical psychology and eating disorders. Inter-rater agreement was evaluated using Fleiss’ kappa (κ = 0.76), indicating substantial agreement. Discrepancies were resolved by consensus. Items included both stigmatizing and counter-stigmatizing formulations and were designed for comprehension by the general population.

#### 2.3.3. Sociodemographic and Experiential Variables

Participants reported age, sex, country of residence, employment in health/social services, education, marital status, employment status, and occupational category.

Experiential variables included:

- Lifetime professional ED diagnosis (multiple options: none, anorexia nervosa, bulimia nervosa, binge-eating disorder, pica, rumination disorder, Avoidant/Restrictive Food Intake Disorder, or none).
- Close family/friend history of ED or obesity.
- Personal history of underweight, overweight, or obesity for ≥3 consecutive months. Classification of individuals as underweight (Body Mass Index, BMI ≥ 18.5), overweight (BMI ≥ 25), or obese (BMI ≥ 30) was based on the World Health Organization [32] criteria widely used in research [33].

These variables were included due to their documented influence on stigma and to examine experiential familiarity effects.

### 2.4. Procedure

Data were collected via Qualtrics (Provo, UT, USA) between February and November 2024. After accessing the study link, participants reviewed an information sheet describing the study aims, procedures, voluntary nature of participation, and data protection measures. Electronic informed consent was obtained prior to participation.

The survey included the stigma item set followed by sociodemographic and experiential questions. Completion time was approximately 15–20 min, and participants could withdraw at any time without penalty. The study protocol was approved by the Ethics Committee of the Jaume I University.

### 2.5. Data Analysis

Descriptive statistics were computed for all stigma items and relevant sociodemographic variables. Inter-rater agreement for item categorization was evaluated using Fleiss’ kappa. Item functioning was examined using item-level descriptive statistics (mean, standard deviation, skewness, and kurtosis) and item response theory (IRT) modeling.

The internal structure of the scale was examined through exploratory and confirmatory factor analysis in two independent subsamples for cross-validation. Factor retention decisions were based on eigenvalues greater than 1.0, inspection of the scree plot, and conceptual interpretability of the factor solution. Internal consistency was assessed using Cronbach’s alpha, with values of 0.70 or higher indicating acceptable reliability [34].

Associations between stigma dimensions and sociodemographic and experiential variables were examined using independent-samples *t* tests or analyses of variance (ANOVAs), as appropriate. Statistical significance was set at *p* < 0.05.

## 3. Results

A total of 2892 individuals accessed the online survey, of whom 2793 provided basic sociodemographic information. Of these, 2268 initiated the stigma questionnaire (reported in the Abstract as the initial analytical sample). After excluding 147 participants (6.5%) who failed embedded attention checks, the final valid sample consisted of 2121 participants, which constituted the primary analytical sample. Comparisons of age, sex, and educational level between participants who completed the stigma questionnaire and those who did not showed no significant differences (all ps > 0.05), indicating that attrition was not systematically related to these variables.

Item-level descriptive statistics analyses were conducted using all participants who passed the control items (*n* = 2121), including those with partially completed responses to the stigma questionnaire. Exploratory and confirmatory factor analyses were performed using complete-case data, including only participants who passed the control items and completed all stigma items (*n* = 1646). This complete-data sample was randomly divided into two independent subsamples for cross-validation purposes: 988 participants were allocated to exploratory factor analysis (EFA), and 658 to confirmatory factor analysis (CFA). Factor retention was determined based on eigenvalues greater than 1.0, inspection of the scree plot, and conceptual interpretability. Internal consistency was evaluated using Cronbach’s alpha, with values ≥ 0.70 considered acceptable. Associations between stigma dimensions and sociodemographic and experiential variables were examined using independent-samples *t* tests or analyses of variance (ANOVAs), with statistical significance set at *p* < 0.05.

The final analytical sample included 2121 participants for descriptive analyses and 1646 for factor analyses. Most participants resided in Spain (96.6%, *n* = 2049). The mean age was 35.7 years (SD = 12.4), ranging from 18 to 78 years. The sample was predominantly female (76.7%, *n* = 1626), followed by male participants (23.1%, *n* = 489) and intersex individuals (0.3%, *n* = 6).

Regarding educational attainment, 34.0% (*n* = 721) reported having completed university-level education, 24.2% (*n* = 513) vocational or technical training, 17.1% (*n* = 363) secondary education or high school, and 2.7% (*n* = 57) primary education.

Employment status was heterogeneous: 51.9% (*n* = 1101) were employed and 25.1% (*n* = 532) were students, with smaller proportions reporting temporary sick leave, unemployment, retirement, or other employment situations.

In terms of occupational category, 9.8% (*n* = 208) were healthcare professionals, 10.4% (*n* = 221) were other professionals, 10.0% (*n* = 212) were students, and 58.8% (*n* = 1247) were classified as belonging to other occupational categories. Regarding body weight history, 41.4% of participants reported maintaining a normal BMI over time, 33.8% reported being overweight, 13.3% reported obesity, and 11.5% reported periods of underweight. A minority reported having received a professional diagnosis of an eating disorder, including anorexia nervosa (2.3%), bulimia nervosa (2.9%), binge-eating disorder (3.2%), avoidant/restrictive food intake disorder (2.0%), pica (0.3%), and rumination disorder (0.3%). With respect to family history, 36.7% reported no close relatives with an eating disorder or obesity, whereas 15.7% reported anorexia nervosa, 10.2% bulimia nervosa, 3.9% binge-eating disorder, 3.1% avoidant/restrictive food intake disorder, 0.2% pica, 0.1% rumination disorder, and 20.1% obesity.

### 3.1. Item Screening and Scale Refinement Based on Distributional Properties

The 66-item questionnaire assessing beliefs and attitudes toward eating disorders was examined for psychometric adequacy. Items, which are presented in Appendix A with their hypothesized theoretical categories, were flagged and removed if they showed:

1. Severe non-normality (|skewness| > 2 or kurtosis > 7),
2. Extreme mean values near the scale endpoints (≈1.0–1.3 or 4.7–5.0),
3. Very low variability (SD < 0.70).

These criteria identified items with floor/ceiling effects or limited discrimination, which can distort factor solutions. The retained 55 items exhibited a broad range of means (M = 1.16–4.88) and SDs (0.51–1.36), reflecting substantial heterogeneity (Table 1). Eleven items displayed extreme distributions (high skewness/kurtosis, means near scale extremes), including broadly consensual statements (e.g., item 32: “Eating disorders can have serious physical health consequences”) or oversimplified beliefs (e.g., item 59: “Eating disorders are basically reduced to anorexia”). These items were removed to reduce noise and enhance factor interpretability. The remaining items retained sufficient variability to capture meaningful differences across stigma domains, including legitimacy, visibility, etiological beliefs, personal responsibility, treatment attitudes, and recovery expectations.

### 3.2. Item Screening and Scale Refinement Based on Exploratory Factor Analysis

Following EFA, additional items were removed due to weak factor loadings, conceptual redundancy, or misalignment with the intended constructs. Accordingly, an exploratory bifactor analysis with the 55 retained after was conducted on the EFA subsample (*n* = 988) using polychoric correlations and the MINRES extraction method. Bifactor solutions specifying one general factor and 2–10 specific factors were evaluated with bifactor rotation. A bifactor model was chosen because the data showed a dominant general factor accounting for most variance (see below).

Initially, a seven-factor bifactor model (one general, six specific) was retained for item evaluation based on model fit, parsimony, and interpretability. The general factor accounted for 65.9% of explained common variance. General-factor loadings were stable across items (|λg| ≥ 0.30). The six specific factors accounted for smaller proportions of variance (4–7%), capturing residual, meaningful content.

Eighteen items with low general-factor loadings (|λg| < 0.30) and negligible common variance (p^2^ < 0.10) were removed (Table 1). The remaining 36 items showed moderate-to-high general-factor loadings (λg = 0.31–0.70) and acceptable communalities, supporting an essentially unidimensional structure with secondary content factors.

### 3.3. Model Fit and Dimensional Structure

As seen in Table 2, the bifactor EFA with the 36 retained items showed progressive RMSEA improvement from 0.060 (1G + 2S) to 0.046 (1G + 10S). TLI remained below conventional cutoffs (0.80–0.88), while BIC indicated the optimal solution was 1 general + 3 specific factors. The general factor accounted for most variance (SS loadings = 9.30), whereas specific factors explained smaller proportions (SS loadings = 0.81–1.17). Several items loaded on both general and specific factors, supporting a bifactor structure dominated by a strong general dimension (Table 3). Overall, findings support a primarily unidimensional scale. Total scores are meaningful, whereas subscale scores should be interpreted cautiously.

### 3.4. Confirmatory Factor Analysis

A CFA was conducted on an independent subsample (*n* = 658) using WLSMV estimation with items as ordered categorical indicators. Two bifactor models were tested: an empirically derived model (1G + 3S) and a theory-driven model (1G + seven content-based specific factors, namely Sociodemographic Stereotypes, Severity and Legitimacy, Personal Responsibility, Visibility and Detectability, and Treatment Beliefs).

As reported in Table 4, the empirically derived model fit well: χ^2^(585) = 853.38, CFI = 0.988, TLI = 0.988, RMSEA = 0.026 (90% CI [0.022, 0.030]), SRMR = 0.053. The theory-driven model also showed excellent fit: χ^2^(561) = 771.65, CFI = 0.991, TLI = 0.990, RMSEA = 0.024 (90% CI [0.020, 0.028]), SRMR = 0.050. Both exceeded conventional criteria for good fit (CFI/TLI ≥ 0.95; RMSEA ≤ 0.06; SRMR ≤ 0.08).

The theory-driven model was preferred for theoretical alignment and slightly superior fit. Despite the subscales, the general factor dominated, indicating total scores are the most robust measure of stigma.

### 3.5. Reliability

General-factor loadings ranged from 0.23 to 0.72 (see Table 5). Specific-factor loadings were generally >0.20; lower values were omitted for clarity.

The general factor showed excellent reliability (α = 0.88; ordinal α = 0.92; ω = 0.80; ω_h_ = 0.87). Specific factors were less reliable, with Sociodemographic Stereotypes (α = 0.59; ω_h_ = 0.25) and Personal Responsibility (α = 0.73; ω_h_ = 0.16) being the most robust. These results support a strong general factor with residual variance accounted for by specific dimensions.

### 3.6. Inter-Rater Agreement in Item Categorization

Four independent judges (C.S.-R., L.D.-S., M.P.-M., M.A.) categorized items into predefined domains. Fleiss’ kappa = 0.78, indicating substantial agreement. Most items achieved perfect agreement; discrepancies were limited to conceptually adjacent categories (e.g., Treatment vs. Resource Allocation). These results support the robustness of the categorical framework and justify consensus-based classification for analyses.

### 3.7. Sociodemographic and Clinical Differences in Total Stigma

Sociodemographic and clinical differences in total stigma are shown in Table 6. The total stigma score ranged from 36 to 146 (M = 67.72, SD = 16.94). Male participants reported higher stigma scores (M = 75.75, SD = 18.18) than female participants (M = 65.50, SD = 15.87), a statistically significant difference, t(1642) = 10.38, *p* < 0.001, Cohen’s d ≈ 0.63. Age was positively correlated with total stigma, r = 0.28, *p* < 0.001, indicating that older participants reported higher stigma. Analysis by age groups (0 = 18–24, 1 = 25–34, 2 = 35–44, 3 = 45+) using one-way ANOVA revealed significant differences, F(3, 1642) = 36.66, *p* < 0.001. Post hoc comparisons using Tukey’s HSD indicated that participants aged 45+ reported significantly higher stigma than all younger groups (all *p* < 0.001).

Occupational differences in stigma scores were also observed, F(3, 1623) = 16.81, *p* < 0.001. Participants in the “other” occupations group (M = 70.03, SD = 17.71) reported higher stigma than healthcare professionals (M = 65.70, SD = 15.42), educators (M = 63.24, SD = 15.02), and students (M = 63.88, SD = 14.26). Participants who reported having ever been underweight, overweight, or obese also differed in stigma, F(3, 1625) = 6.63, *p* < 0.001, with underweight (M = 64.28, SD = 14.33) reporting lower stigma than overweight (M = 69.65, SD = 18.43) and normoweight (M = 66.49, SD = 16.11) participants. Having a personal history of a diagnosed eating disorder was associated with lower stigma (M = 65.07, SD = 16.28) compared to participants without a diagnosis (M = 67.91, SD = 16.90), t(1627) = 2.05, *p* = 0.04. Similarly, participants reporting no close relatives with eating disorders or obesity had higher stigma (M = 69.45, SD = 16.77) than those with affected relatives (M = 65.85, SD = 16.75), t(1627) = 4.33, *p* < 0.001.

## 4. Discussion

The present study provides a comprehensive psychometric evaluation of a novel instrument designed to assess societal stigma toward eating disorders and obesity within the Spanish context. The findings offer robust evidence for the scale’s internal structure, reliability, and content validity, while underscoring the pervasive influence of stigma on public perceptions of these conditions [1,2,3,4]. Overall, the results support the conceptualization of stigma as a multidimensional construct dominated by a strong general factor, consistent with contemporary theoretical models of health-related stigma [11,12]. Importantly, the scale development process resulted in a psychometrically robust yet parsimonious instrument, combining conceptual breadth with practical brevity, enhancing its feasibility for research, clinical, and public health applications. These findings are also consistent with research on eating disorder mental health literacy, which shows that limited knowledge and persistent myths about eating disorders are associated with higher stigma, particularly among male and adolescent populations [10]. The strong general stigma factor observed may partly reflect broad deficits in public understanding of eating disorders and obesity, reinforcing the need for educational interventions that directly target misinformation alongside moralized attitudes.

The initial item reduction process highlighted the importance of removing statements exhibiting severe non-normality, floor/ceiling effects, or low variability. These psychometric characteristics have long been recognized as problematic for factor analysis, as they can distort inter-item correlations and inflate factor solutions [35]. In our sample, items reflecting socially or morally consensual beliefs, such as acknowledgment of serious health consequences of eating disorders, were frequently flagged. While these items may be theoretically relevant, their extreme distributions limited their discriminatory power, supporting their exclusion to ensure factorial clarity. The final 36-item pool retained adequate variability across domains, enabling meaningful differentiation between individuals with varying levels of stigmatizing attitudes, while maintaining a compact structure that balances psychometric precision with respondent burden.

Exploratory factor analysis further confirmed the dominance of a general stigma factor, accounting for 65.9% of explained common variance. This finding aligns with prior research indicating that societal stigma toward mental and physical health conditions is often underpinned by overarching negative attitudes, which transcend specific content domains [12,28]. Recent experimental work further suggests that stigma toward disordered eating may be relatively insensitive to diagnostic labels, with similar levels of weight-based and mental illness stigma observed when behaviors are attributed to binge-eating disorder versus food addiction [36]. This supports the present finding of a dominant general stigma factor and suggests that societal responses may be driven more by moralized interpretations of eating behavior and body weight than by nuanced diagnostic distinctions. The secondary specific factors captured residual variance related to content-specific dimensions, such as severity, personal responsibility, visibility, and treatment beliefs [5,6,29,30].

The prominence of responsibility-related content aligns with emerging perspectives that frame binge-eating disorder and obesity-related stigma as rooted in broader systems of oppression and moral regulation of bodies. Expert consensus highlights stigmatization, trauma, economic precarity, and social messaging as central contributors to binge-eating pathology [13], underscoring the importance of measuring stigma not only as an individual attitude but as a structural and societal phenomenon. However, the proportion of variance explained by these factors was relatively small (4–7%), highlighting the psychometric primacy of the general factor. Consequently, the total score of the instrument emerges as the most robust and meaningful measure of stigma, whereas subscale scores, while potentially informative descriptively, should be interpreted cautiously. This structure supports the use of a single, efficient global stigma score for large-scale assessment and monitoring purposes.

Confirmatory factor analysis corroborated the bifactor structure identified in the EFA. Both the empirically derived model and the theory-driven model demonstrated excellent fit according to conventional indices (CFI/TLI ≥ 0.95, RMSEA ≤ 0.06, SRMR ≤ 0.08), indicating that the scale reliably captures latent stigma constructs. Preference was given to the theory-driven model for its theoretical alignment and slightly superior fit, highlighting the value of grounding psychometric models in conceptual frameworks rather than relying solely on statistical criteria. Nonetheless, the dominance of the general factor reinforces the notion that societal stigma toward eating disorders and obesity operates primarily as a broad evaluative construct, with secondary content-specific dimensions contributing residual but meaningful information. Therefore, the findings support the use of a total stigma score as the primary index. Subscale scores should be interpreted cautiously and are best considered descriptive complements rather than independent constructs. This configuration allows the instrument to remain theoretically rich while operationally streamlined.

The reliability analyses provide additional evidence for the robustness of the general factor. Internal consistency was excellent across indices (α = 0.88, ordinal α = 0.92, ω_h_ = 0.87), demonstrating that the total score can be confidently interpreted as a reliable indicator of overall stigma. In contrast, the specific factors displayed variable reliability. The relatively low reliability of some specific factors suggests that these dimensions may not be equally stable across populations, and further validation is needed before their independent use is recommended. While the subscales may offer descriptive nuance for research or intervention purposes, their psychometric limitations warrant caution in clinical or policy-oriented applications. This further supports the use of the brief total score as the primary index of stigma.

From a theoretical perspective, the findings reinforce contemporary models that conceptualize stigma as a multifaceted, socially mediated process. The strong general factor observed is consistent with the Health Stigma and Discrimination Framework, which posits that stigma operates through labeling, stereotyping, moral evaluation, and discrimination, embedded within broader social structures of power and inequity [12]. The persistence of a dominant general factor across EFA and CFA suggests that societal attitudes toward eating disorders and obesity are largely generalized, reflecting overarching negative evaluations rather than solely condition-specific beliefs [1,2]. However, the presence of meaningful residual variance in specific content domains indicates that targeted interventions could address particular aspects of stigma, such as misconceptions about treatment, personal responsibility, or visibility [3,4,29,30].

### Sociodemographic and Clinical Differences in Stigma

Consistent with prior literature, sociodemographic factors were significantly associated with stigma scores. Male participants reported higher stigma than female participants, with the difference moderate-to-large in magnitude (Cohen’s d ≈ 0.63). Age was positively correlated with stigma, and participants aged 45 and older exhibited significantly higher stigma than younger adults. These results suggest that cultural norms and generational attitudes may shape stigmatizing beliefs, with older and male participants potentially endorsing more traditional stereotypes about body weight and eating behavior. Occupational status was also linked to stigma, with individuals in the “other” occupations group reporting higher stigma than healthcare professionals, educators, or students. This pattern may reflect greater exposure to training or knowledge about health conditions among professionals and students, highlighting the protective role of education and professional experience against stigmatizing attitudes. All these findings are consistent with recent stigma research in psychosis [37] and points to targets of stigma societal prevention programs.

Clinical variables were similarly relevant. Participants with a personal history of a diagnosed eating disorder reported lower stigma compared to those without such a history [3,28], consistent with research indicating that personal experience can mitigate negative attitudes through increased empathy and perspective-taking. Similarly, participants reporting close relatives affected by eating disorders or obesity demonstrated lower stigma than those without affected relatives, supporting the notion that familiarity reduces stereotyping. BMI history was also associated with stigma: underweight individuals reported lower stigma than normoweight or overweight participants, while overweight participants reported the highest stigma. These findings suggest that personal experiences with body weight influence perceptions of responsibility, severity, or social acceptability, reinforcing the importance of considering both experiential and demographic factors in anti-stigma interventions.

The implications of these findings are particularly salient in Spain, where eating disorders are estimated to affect 1–5% of the population [14,15,16] and obesity affects over half of the population [18]. Stigmatizing beliefs—such as attributions of laziness or moral weakness—can exacerbate psychological distress, impede help-seeking, and reduce engagement with evidence-based interventions [5,6,9]. The validated instrument developed here enables systematic measurement of these societal attitudes, providing critical insights for public health strategies, anti-stigma campaigns, and research examining the consequences of stigma on mental and physical health outcomes [19,21,23].

The study also highlights methodological considerations for stigma research. First, the inclusion of individuals with lived experience of eating disorders and obesity enhances ecological validity and allows examination of stigma across experiential levels, as recommended by best practice task forces [11,12]. Second, the use of embedded control items to identify inattentive responding ensured high-quality data, strengthening the reliability of psychometric conclusions. Finally, the multistage development process, integrating expert judgment, content validation, EFA, CFA, and reliability assessment, exemplifies best practices in scale construction and supports the broader adoption of the instrument in Spanish-speaking contexts.

Several limitations warrant consideration. The use of convenience and snowball sampling may limit the representativeness of the sample, and the overrepresentation of women may have influenced observed stigma levels, given known gender differences in stigma attitudes. The cross-sectional design precludes causal inferences regarding the impact of stigma on behavior or health outcomes, highlighting the need for longitudinal studies to examine temporal dynamics and intervention effects. Additionally, the exclusive reliance on self-report data collected at a single time point raises the possibility of shared method variance. While the instrument demonstrates robust psychometric properties, further validation in fully independent samples, including clinical populations and cross-cultural contexts would confirm the stability of the factor structure, strengthen its applicability and ensure sensitivity to cultural nuances in stigma expression. Finally, although necessary for psychometric reasons, the removal of highly skewed items may have led to the exclusion of socially important but widely endorsed beliefs, potentially narrowing the conceptual breadth of the construct.

In conclusion, the present study offers a psychometrically robust instrument for assessing societal stigma toward eating disorders and obesity in Spain, capturing both a dominant general stigma factor and meaningful content-specific dimensions. The findings underscore the pervasiveness of stigma, its broad evaluative nature, and the influence of sociodemographic and clinical factors on its expression. Importantly, the instrument fills a critical gap in Spanish-language research, providing a tool for the systematic evaluation of stigma and informing interventions aimed at reducing blame, moralization, and social prejudice. By integrating multidimensional stigma domains within a robust bifactor structure and providing validation in a Spanish-speaking context, this instrument advances existing measures and offers a practical tool for large-scale public health surveillance and evaluation of anti-stigma interventions. Future research should explore the relationship between stigma, help-seeking, treatment engagement, and health outcomes, as well as the efficacy of public health campaigns and clinical interventions designed to promote compassion, understanding, and equitable access to care for individuals affected by eating disorders and obesity.

## Figures and Tables

**Table 1:** Item Descriptive Statistics, Conceptual Domains, and Retention Decisions after the Exploratory Factor Analysis (*n* = 2121).

	Item Reduction Phase 1				Item Reduction Phase 2					
Item	Mean	sd	Skew	Kurtosis	Lambda	h^2^	*p* ^2^	Complexity	Decision	Reason
1	3.04	1.27	−0.03	−1.11	0.359	3.521	0.029	2.7	Retain	
2	2	1.17	1.07	0.12	0.556	3.095	0.1	3.33	Retain	
3	4.88	0.51	−5.53	34.84					Remove	ceiling effect
4	4.79	0.57	−3.69	16.96					Remove	ceiling effect
5	3.99	1.08	−1.05	0.43	−0.257	11.384	0.008	1.19	Remove	Weak indicator
6	1.49	0.89	1.94	3.31	0.364	4.023	0.031	2.2	Retain	
7	4.39	0.84	−1.55	2.51	−0.163	11.806	0.004	1.16	Remove	Weak indicator
8	3.88	0.92	−0.62	0.25	−0.037	6.221	0	1.32	Remove	Weak indicator
9	3.51	1.22	−0.55	−0.72	−0.095	2.096	0.004	2.8	Remove	Weak indicator
10	2.24	1.3	0.7	−0.79	0.578	3.691	0.092	2.7	Retain	
11	1.39	0.84	2.35	5.04	0.61	3.031	0.12	3.53	Retain	
12	2.51	1.35	0.31	−1.26	0.555	3.883	0.081	2.46	Retain	
13	2.31	1.23	0.69	−0.59	0.525	3.029	0.084	3.37	Retain	
14	3.94	1.11	−1.01	0.27	0.1	5.408	0.002	1.38	Remove	Weak indicator
15	2.89	1.28	−0.01	−1.22	0.119	2.248	0.009	2.69	Remove	Weak indicator
16	3.14	1.1	−0.41	−0.45	0.093	2.235	0.003	2.28	Remove	Weak indicator
17	2.33	1.16	0.74	−0.38	0.309	3.321	0.032	2.86	Retain	
18	1.72	1.16	1.64	1.6	0.595	3.037	0.135	3.63	Retain	
19	1.41	0.8	2.3	5.14	0.618	3.055	0.1	3.39	Retain	
20	1.67	1.02	1.51	1.22	0.639	3.112	0.108	3.41	Retain	
21	1.42	0.8	2.23	5.23	0.405	3.431	0.057	2.77	Retain	
22	1.22	0.64	3.61	14.54					Remove	floor effect
23	1.3	0.72	2.82	8.08					Remove	floor effect
24	4.2	0.96	−1.5	2.22	0.022	4.571	0	1.47	Remove	Weak indicator
25	1.27	0.71	3.12	10.35					Remove	floor effect
26	2.98	1.31	0.08	−0.97	0.349	3.408	0.027	2.82	Retain	
27	1.63	0.98	1.61	1.85	0.57	2.925	0.122	3.62	Retain	
28	1.73	1.06	1.41	1.01	0.575	3.04	0.118	3.51	Retain	
29	2.63	1.31	0.23	−1.17	0.266	8.977	0.007	1.27	Remove	Weak indicator
30	2.37	1.23	0.32	−1.1	0.185	2.126	0.006	2.52	Remove	Weak indicator
31	2.39	1.25	0.35	−1.14	0.285	4.964	0.017	1.76	Remove	Weak indicator
32	4.74	0.82	−3.72	13.45					Remove	ceiling effect
33	2.1	1.18	0.87	−0.31	0.561	2.976	0.111	3.54	Retain	
34	3.1	1.27	−0.39	−1.06	0.147	2.743	0.005	1.95	Remove	Weak indicator
35	1.99	1.12	0.86	−0.33	0.637	3.015	0.129	3.6	Retain	
36	1.86	1.02	0.78	−0.63	0.276	9.971	0.008	1.26	Remove	Weak indicator
37	4.22	0.86	−1.53	3.17	0.004	5.421	0	1.39	Remove	Weak indicator
38	1.57	0.89	1.76	2.75	0.447	8.503	0.022	1.34	Retain	
39	1.21	0.63	3.72	15.34					Remove	floor effect
40	1.4	0.81	2.22	4.48	0.587	3.002	0.118	3.52	Retain	
41	1.74	1.07	1.4	1.08	0.492	3.068	0.09	3.27	Retain	
42	1.73	1.13	1.41	0.81	0.592	2.933	0.122	3.62	Retain	
43	1.16	0.54	4.3	21					Remove	floor effect
44	1.47	0.96	2.24	4.35	0.455	3.152	0.071	3.14	Retain	
45	2.77	1.36	0.14	−1.3	0.364	3.659	0.039	2.49	Retain	
46	1.55	0.88	1.67	2.24	0.585	3.111	0.118	3.46	Retain	
47	1.65	1.13	1.64	1.54	0.31	4.507	0.024	1.99	Retain	
48	1.98	1.18	0.92	−0.41	0.416	5.54	0.028	1.53	Retain	
49	1.49	0.77	1.74	3.04	0.181	4.649	0.005	1.53	Remove	Weak indicator
50	1.87	1.04	1.09	0.44	0.398	5.586	0.028	1.66	Retain	
51	2.13	1.15	0.62	−0.84	0.227	4.127	0.011	1.62	Remove	Weak indicator
52	2.86	1.29	−0.1	−1.3	0.416	6.91	0.025	1.47	Retain	
53	4.32	0.95	−1.59	2.27	−0.095	2.858	0.005	1.87	Remove	Weak indicator
54	2.13	1.15	0.69	−0.44	0.318	4.012	0.028	2.2	Retain	
55	1.3	0.77	2.86	8.01					Remove	floor effect
56	1.84	1.14	1.08	−0.15	0.516	3.133	0.088	3.24	Retain	
57	1.49	0.89	2.15	4.51	0.612	2.993	0.126	3.59	Retain	
58	1.45	0.74	1.81	3.32	0.542	3.563	0.087	2.65	Retain	
59	1.17	0.56	3.96	17.63					Remove	floor effect
60	1.32	0.71	2.5	6.34					Remove	floor effect
61	1.53	0.99	2.01	3.19	0.699	3.058	0.162	3.76	Retain	
62	1.44	0.86	2.02	3.3	0.418	10.133	0.014	1.24	Retain	
63	2.97	1.25	−0.37	−1.25	0.135	2.76	0.004	1.91	Remove	Weak indicator
64	1.97	1.05	0.89	−0.05	0.544	3.028	0.098	3.4	Retain	
65	1.57	0.89	1.5	1.5	0.303	4.516	0.032	1.88	Retain	
66	3.66	1.32	−0.55	−0.96	0.016	7.677	0.003	1.28	Remove	Weak indicator

Note. Retention decisions were based on both Classical Test Theory criteria (e.g., floor/ceiling effects and distributional properties) and Exploratory Factor Analysis (EFA) results (e.g., factor loadings, stability, and conceptual coherence). Weak indicator = item poorly reflects the general stigma factor.

**Table 2:** Fit indices for bifactor EFA models with one general factor and 2–10 specific factors (*n* = 988).

Model	Total Factors	RMSEA	90% CI RMSEA	TLI	BIC
1G + 2 Specific	3	0.06	[0.057, 0.062]	0.8	−1248.67
1G + 3 Specific	4	0.056	[0.053, 0.058]	0.83	−1391.07
1G + 4 Specific	5	0.054	[0.051, 0.057]	0.84	−1384.66
1G + 5 Specific	6	0.052	[0.049, 0.055]	0.85	−1380.23
1G + 6 Specific	7	0.05	[0.047, 0.052]	0.86	−1385.97
1G + 7 Specific	8	0.049	[0.046, 0.052]	0.87	−1317.01
1G + 8 Specific	9	0.048	[0.045, 0.051]	0.87	−1234.75
1G + 9 Specific	10	0.047	[0.043, 0.050]	0.88	−1180.10
1G + 10 Specific	11	0.046	[0.042, 0.049]	0.88	−1111.96

Note. approximation method = minimum residuals (MINRES) with oblimin rotation. Analyses based on polychoric correlations. Low BIC indicates b-fitting and most parsimonious model.

**Table 3:** Standardized Factor Loadings for the Bifactor EFA Solution (1 General and 3 Specific Factors) (*n* = 988).

Item	General Factor	Specific Factor 1	Specific Factor 2	Specific Factor 3
1	0.31	0.36	—	—
2	0.54	—	—	—
6	0.39	—	—	—
10	0.52	—	0.36	—
11	0.59	—	—	—
12	0.5	—	0.44	—
13	0.48	—	—	—
17	0.33	—	—	—
18	0.6	—	—	—
19	0.58	0.48	—	—
20	0.6	0.54	—	—
21	0.41	0.33	—	—
26	0.31	—	—	—
27	0.57	—	—	—
28	0.56	—	—	—
33	0.54	—	—	—
35	0.59	—	—	—
38	0.47	—	—	—
40	0.59	—	—	0.54
41	0.52	—	—	—
42	0.58	—	—	—
44	0.45	—	—	0.37
45	0.34	—	—	—
46	0.61	—	—	—
47	0.38	—	—	—
48	0.46	—	—	—
50	0.43	—	—	—
52	0.38	—	0.33	—
54	0.4	—	—	—
56	0.47	—	—	—
57	0.63	—	—	—
58	0.57	—	—	—
61	0.73	—	—	—
62	0.5	—	—	—
64	0.61	—	—	—
65	0.39	—	—	—

The general factor reflects overall stigma, whereas the specific factors represent residual groupings after controlling for the general factor. Cross-loadings are expected and appropriate in bifactor models.

**Table 4:** Standardized Factor Loadings for the Theory-Driven Bifactor CFA Model (*n* = 658).

Item	G	F1	F2	F3	F4	F5
1	0.24	0.23	—	—	—	—
2	0.53	—	—	—	—	—
6	0.37	—	—	—	—	−0.21
10	0.53	—	—	0.42	—	—
11	0.62	—	—	—	—	—
12	0.53	—	—	0.34	—	—
13	0.47	—	—	—	0.46	—
17	0.38	—	—	—	—	—
18	0.72	—	—	—	—	—
18	0.72	—	—	—	—	—
19	0.43	0.66	—	—	—	—
20	0.44	0.84	—	—	—	—
21	0.48	0.4	—	—	—	—
26	0.23	—	—	—	—	—
27	0.61	—	—	0.31	—	—
28	0.62	—	—	—	—	—
33	0.59	—	—	—	—	—
35	0.62	—	—	—	—	—
38	0.45	—	—	—	—	—
40	0.61	—	—	—	—	—
41	0.56	—	—	—	—	—
42	0.59	—	—	0.22	—	—
44	0.52	—	—	—	—	—
45	0.41	—	—	—	—	0.31
46	0.64	—	—	—	0.25	—
47	0.35	—	0.29	—	—	—
48	0.41	—	—	—	—	—
50	0.43	—	—	—	—	—
52	0.36	—	—	0.35	—	—
54	0.34	—	—	0.2	—	—
56	0.57	—	−0.22	—	—	—
57	0.64	—	—	—	—	—
61	0.69	—	—	—	—	—
64	0.53	—	0.51	—	—	—
65	0.48	—	—	—	—	—

Note. Standardized factor loadings are shown for the general factor and five specific factors in the theory-driven bifactor CFA model. Loadings < |0.20| are represented as dashes (—). G, General factor; F1, Sociodemographic; F2, Severity & Legitimacy; F3, Personal Responsibility; F4, Visibility & Detectability; F5, Treatment.

**Table 5:** Reliability values for the theoretical bifactor CFA model (*n* = 658).

Factor	α	Ordinal α	ω	ω_h_
General factor	0.88	0.92	0.8	0.87
Sociodemographic stereotypes	0.59	0.71	0.35	0.25
Severity and legitimacy	0.7	0.81	0.07	0.03
Personal responsibility	0.73	0.81	0.29	0.16
Visibility and detectability	0.56	0.68	0.15	0.09
Treatment beliefs	0.37	0.5	0.04	0.03

Note. ω_h_ = omega hierarchical, reflecting reliable variance unique to each factor after controlling for the general factor.

**Table 6:** Total stigma scores across demographic and clinical categories.

Variable	Category	*n*	%	M (SD) Stigma
Sex	Male	490	23.1	75.75 (18.18)
	Female	1627	76.7	65.50 (15.87)
Age group	18–24	445	21	63.65 (14.88)
	25–34	339	16	64.90 (16.16)
	35–44	424	20	67.83 (15.24)
	45+	382	18	74.66 (19.26)
Occupation	Other	976	58.8	70.03 (17.71)
	Healthcare	222	13.4	65.70 (15.42)
	Professional	235	14.2	63.24 (15.02)
	Student	227	13.7	63.88 (14.26)
BMI history	Underweight	190	11.5	64.28 (14.33)
	Normoweight	684	41.4	66.49 (16.11)
	Overweight	559	33.8	69.65 (18.43)
	Obesity	220	13.3	68.94 (16.27)
Diagnosed eating disorder	Any	243	14.7	65.07 (16.28)
Family eating disorder/obesity	Any	1312	63.3	65.85–69.45

## Data Availability

Data will be shared upon request to the corresponding author.

## Appendix A. Complete List of Items

**Item Number**	**Item Description**	**Theoretical Basis**
1	Eating disorders such as anorexia, bulimia, or binge-eating disorder are very rare in old age.	Sociodemographic stereotypes
2	Worrying and thinking a lot about food and weight cannot be that harmful.	Severity and legitimacy
3	A severe eating disorder can lead to death.	Severity and legitimacy
4	Cultural ideals of beauty strongly influence the development of eating disorders.	Etiological beliefs
5	Excessive exercise may conceal an eating disorder.	Visibility and detectability
6	Eating disorders should be kept secret within the family.	Treatment
7	Psychological factors such as excessive perfectionism and impulsivity increase the risk of developing an eating disorder.	Etiological beliefs
8	If a person has an eating disorder, it is easy for them to end up developing at least one more.	Chronicity and recovery
9	Having an eating disorder makes you permanently vulnerable to these disorders.	Chronicity and recovery
10	People with overweight or obesity have little willpower.	Personal responsibility and moral judgment
11	Anorexia and bulimia are a fad.	Severity and legitimacy
12	People who eat to regulate their emotions are psychologically weak.	Personal responsibility and moral judgment
13	If someone around me were binge eating and then vomiting or using laxatives to compensate, I would surely have noticed.	Visibility and detectability
14	There is always a psychological reason behind an eating disorder or obesity.	Etiological beliefs
15	Obesity problems and eating disorders such as anorexia, bulimia, and binge-eating disorder are never completely resolved.	Chronicity and recovery
16	Certain eating disorders, such as bulimia and anorexia, are more frequent just before and during summer.	Sociodemographic stereotypes
17	People with an eating disorder know that they have a problem.	Visibility and detectability
18	Eating disorders are not concerning until there is excessively low or high weight.	Severity and legitimacy
19	Eating disorders are women’s issues.	Sociodemographic stereotypes
20	Eating disorders are adolescent issues.	Sociodemographic stereotypes
21	Obesity is very uncommon in childhood and mainly occurs in old age.	Sociodemographic stereotypes
22	There is nothing wrong with using laxatives, vomiting, using diuretics, or exercising excessively to maintain weight.	Severity and legitimacy
23	Certain eating disorders are problems of wealthy people.	Sociodemographic stereotypes
24	Low self-eem greatly increases the risk of obesity or an eating disorder.	Etiological beliefs
25	People with anorexia cope well because they do not feel hungry.	Severity and legitimacy
26	Adults rarely eat or lick non-nutritive and unusual substances such as paper, ice, hair, or soil.	Sociodemographic stereotypes
27	People who have an eating disorder or overweight are to blame for their problem.	Personal responsibility and moral judgment
28	People with an eating disorder are simply going through a phase.	Severity and legitimacy
29	People with obesity or an eating disorder must be unhappy.	Sociodemographic stereotypes
30	Certain eating disorders, such as bulimia and binge-eating disorder, are more frequent at Christmas.	Sociodemographic stereotypes
31	In reality, everyone has some type of eating disorder nowadays.	Severity and legitimacy
32	Eating disorders can have serious consequences for the physical health of those who suffer from them.	Severity and legitimacy
33	In families with healthy lifestyles, eating disorders and obesity problems do not occur.	Personal responsibility and moral judgment
34	Eating disorders are more common among people who practice certain sports or professions such as models and dancers.	Sociodemographic stereotypes
35	Only certain people are sufficiently vulnerable to develop eating disorders.	Sociodemographic stereotypes
36	Eating disorders are determined by genes.	Etiological beliefs
37	Stress greatly increases the likelihood of developing an eating disorder.	Etiological beliefs
38	There is little hope that a person with an eating disorder or obesity will improve.	Chronicity and recovery
39	Too many public resources should not be spent on treating people with anorexia, bulimia, binge-eating disorder, or obesity.	Treatment
40	I find it hard to understand how someone could end up developing an eating disorder or obesity.	Severity and legitimacy
41	Everyone binges, and that is not a problem.	Severity and legitimacy
42	People who induce vomiting or use laxatives to purge themselves are mentally ill.	Personal responsibility and moral judgment
43	A person with anorexia is cured by gaining weight.	Severity and legitimacy
44	I would find it difficult to empathize with someone with an eating disorder or obesity.	Treatment
45	Those who suffer the most from eating disorders such as anorexia, bulimia, and binge-eating disorder are the families.	Treatment
46	A person who binge eats can be easily identified just by their appearance.	Visibility and detectability
47	Purging by using, for example, laxatives, diuretics, vomiting, or excessive exercise are effective methods if you want to avoid gaining weight.	Severity and legitimacy
48	Eating disorders such as anorexia are the result of diets that have gone too far.	Etiological beliefs
49	People with overweight or obesity cannot do anything to avoid being overweight.	Personal responsibility and moral judgment
50	People who lose weight rapidly have an eating disorder.	Visibility and detectability
51	Parents are responsible for causing eating-related problems in their children.	Personal responsibility and moral judgment
52	Obesity is the result of certain lifestyles chosen by the individual.	Personal responsibility and moral judgment
53	People with eating disorders such as anorexia, bulimia, and binge-eating disorder have a distorted body image.	Etiological beliefs
54	People have the right to have overweight or an eating disorder if they want to.	Personal responsibility and moral judgment
55	Eating disorders are cured through strict discipline.	Treatment
56	Eating disorders such as anorexia, bulimia, or binge-eating disorder are ways of seeking attention.	Severity and legitimacy
57	People with a normal weight cannot have an eating disorder.	Visibility and detectability
58	Eating disorders are rare.	Severity and legitimacy
59	Eating disorders are basically limited to anorexia.	Severity and legitimacy
60	People with an eating disorder are very thin.	Visibility and detectability
61	Recovering from an eating disorder is very easy; it depends on the person’s willpower.	Personal responsibility and moral judgment
62	Certain eating disorders are problems of people with low socioeconomic status.	Sociodemographic stereotypes
63	Eating disorders are basically a consequence of the media and social networks.	Etiological beliefs
64	It is completely normal to want to control weight in a very strict way.	Severity and legitimacy
65	Praising the physical appearance of a person with an eating disorder helps to cure them.	Treatment
66	Dieting has nothing to do with the development of an eating disorder.	Etiological beliefs
